# Antibacterial Efficacy of Aqueous Ozone in Root Canals Infected by *Enterococcus faecalis*

**DOI:** 10.5812/jjm.11411

**Published:** 2014-07-01

**Authors:** Ihsan Hubbezoglu, Recai Zan, Tutku Tunc, Zeynep Sumer

**Affiliations:** 1 Department of Restorative Dentistry, Faculty of Dentistry, Cumhuriyet University, Sivas, Turkey; 2 Department of Endodontics, Faculty of Dentistry, Cumhuriyet University, Sivas, Turkey; 3 Department of Microbiology, Faculty of Medicine, Cumhuriyet University, Sivas, Turkey

**Keywords:** Ozone, Disinfection, Root Canal, *Enterococcus faecalis*

## Abstract

**Background:**

In endodontics, the elimination of resistant bacteria such as *Enterococcus faecalis* plays an important role for treatment success in root canals. Therefore, new alternative irrigants (instead of sodium hypochlorite) have been researched to achieve ideal endodontic treatment.

**Objectives:**

The aim of the present study was to evaluate and to compare the antibacterial effect of aqueous ozone with different concentrations and techniques of application (manual and ultrasonic) against *E.* *faecalis* in human root canals.

**Patients and Methods:**

Eighty single-root mandibular premolar teeth were selected, prepared and sterilized. *E.* *faecalis* was incubated in the root canals and kept at 37°C for 24 h. The teeth were divided into four main groups each has 20 members: NaOCl (positive control) group; 8 ppm aqueous ozone group; 12 ppm aqueous ozone group; and 16 ppm aqueous ozone group. While half of the specimens were disinfected with aqueous ozone by manual technique, the other half was disinfected with the aqueous ozone by ultrasonic technique. Conventional irrigation technique was simultaneously applied with ultrasonic vibration that was produced by VDW.ULTRA device. The disinfection procedures were performed for 180 s to ensure standardization of all the working groups. Paper points (placed in the root canals before and after the disinfection procedures) were transferred to Eppendorf tubes containing 0.5 mL of brain heart infusion broth. Then, 50 μL of the suspension was inoculated onto broth agar media. Microbial colonies were counted, and the data were evaluated statistically using 2-way analysis of variance (ANOVA) and Tukey tests.

**Results:**

Although the antibacterial effect of 16 ppm aqueous ozone using a manual technique had an insufficient effect, its ultrasonic application technique resulted in complete disinfection in the root canals.

**Conclusions:**

The bactericidal activity of high concentration of aqueous ozone combined with ultrasonic application technique showed efficacy similar to that of 5.25% NaOCl in root canals.

## 1. Background

In endodontics, root canal irrigation is an integral part of preparation, which plays an important role in disinfection and removal of the debridement (1). The ideal irrigant should be strongly antimicrobial, able to dissolve the necrotic tissues, the remaining organic tissues, and be non-toxic to the periapical tissues if extruded through the apex (2). For instance, sodium hypochlorite, NaOCl, has been widely used as an irrigation solution for the chemomechanical treatment of root canals because of its solvent activity on both necrotic and vital tissues. Moreover, NaOCl demonstrates stronger antimicrobial efficacy in root canals than other irrigation solutions (3). However, one of the most important disadvantages of NaOCl is its strong toxicity to the periapical tissues when it extrudes through the apex (4).

Not only irrigation solution but also the irrigation technique plays a crucial role in successful endodontic treatment. For example, the conventional irrigation technique cannot eliminate some microorganisms that have acquired resistance to irrigation solutions (5). Therefore, new irrigation techniques have emerged to achieve ideal root canal disinfection. For example, the conventional irrigation technique has been combined with ultrasonication to obtain a higher antibacterial effect compare to the conventional irrigation techniques (6-8).

*Enterococcus faecalis* is the most commonly isolated and persistent bacteria in root canals. *E.* *faecalis* is a Gram-positive facultative anaerobe that can attach the dentin and to invade dentinal tubules (9). Moreover, it shows high bacterial resistance to several irrigation solutions and medications used in endodontics. Therefore, *E.* *faecalis* can survive for a long time in the root canals (10). One of the new generations of the disinfectant agents is ozone; a powerful oxidizing agent used to eliminate bacteria in root canals (11). Recent investigations of aqueous ozone have indicated that it is a powerful antimicrobial agent against oral pathogens. This suggests that aqueous ozone at different doses might eliminate the oral resistant microorganisms too (7, 12, 13). One of the crucial properties of aqueous ozone is its nontoxicity to oral cells in vitro.

 On the other hand, it is less toxic than all other known antiseptics (14). However, the most important disadvantage of aqueous ozone is its unstable concentration in a long time. Consequently, aqueous ozone should be used as soon as possible after obtaining the ozone generator (15). These properties indicated that aqueous ozone could be beneficial in many branches of dentistry, and its use has been recommended by some researchers for the treatment of endodontic infections (7, 15).

## 2. Objectives

The present study aimed to evaluate and to compare the antibacterial effect of aqueous ozone with different concentrations and techniques of application (manual and ultrasonic) against *E.* *faecalis* in human root canals. Moreover, the resulted data were compared regarding the antibacterial efficacy of NaOCl (positive control) groups for the sterilization of root canals infected by *E.* *faecalis*.

## 3. Patients and Methods

### 3.1. Samples Preparation 

This study was approved by the Local Ethics Committee on Human Research of Cumhuriyet University (2011/016), and the informed consents were obtained from the patients prior to the research. In the present study, we used 80 single-rooted mandibular permanent premolar teeth that were freshly extracted for orthodontic or periodontal reasons (without caries and restorations). Digital radiographs of teeth were taken from the buccal and proximal directions to determine the number and morphology of their canals. After having been cleaned of residues, the freshly extracted teeth were kept at +4°C in 0.9% saline solution during the whole study.

Below the level of the cementoenamel junction, the coronal portions of the teeth were cut using sterile diamond discs under cooling water to obtain a 14-16 mm length for each root. Then the root canals were entered with a 15-number K-File (Mani Inc., Tochigi, Japan) hand tool, and the path of the canal was determined. The tip of the file was transmitted to measure the length of each canal until it became visible in the apical foramen. Then 1 mm was withdrawn from the measured length. The root canals were shaped with a ProTaper (Dentsply, Tulsa Endodontics, OK, USA) rotary NiTi instruments using the crown-down method by the electric motor (Dentaports DP-ZX, J. Morita Mfg, Corp, Kyoto, Japan).

First, the coronal third of the root was expanded with an SX file. Next, the median third of the roots was reached with a S1 or S2 file. The F1, F2, and F3 files were consecutively applied to shape the apical third of the canals. The canals were irrigated with 1 ml of 5.25% NaOCl solution after the use of each file. The roots were irrigated with 17% EDTA, 5.25% NaOCl, and distilled water consecutively for 5 min to remove the smear layer that formed during the root canal preparation. Then they were dried with paper points. Next, a 3-fold nail polish (L'Oreal Jet-Set Diamond, Paris, France) was applied to all root surfaces of the teeth, including the root tips. After rubber caps were embedded, the teeth were sterilized using ethylene oxide. Finally, the caps were placed in bottles. The bottles were already placed in an autoclave (Melag, Euroklav 23V-S, Germany) for 20 min at 121°C to ensure sterilization.

### 3.2. Microbiologic Procedures

*E. faecalis* (ATCC 29212) strains were cultured in the liquid nutrient media (brain heart infusion broth, [Acumedia Manufacturers, Inc. Lansing, Michigan, USA]) and incubated at 37°C for 24 h. Prior to each experiment, 0.5 McFarland turbidity was set with the Cristalspec^TM^ device and McFarland standard number 0.5 was used to improve blood agar plates to obtain bacterial growth to the amount of 1.5 X 108 colonies forming unit (CFU/mL). 

Afterwards, 10 μL of the bacterial culture were transferred to the mechanically expanded lumen of the root canal using a sterile micropipette and kept at 37°C for 24 h. In order to control bacterial growth, sterile paper points (Dentsply Maillefer) were placed in the root canals inoculated with bacteria to control bacterial growth. The paper points left for 5 min in the root canals, soaked with the broth. Then the paper points were placed into sterile Eppendorf tubes containing 0.5 mL brain heart infusion broth (Merck, Germany). After 15 minutes, 50 μL of the liquid medium was taken with a sterile micropipette from the Eppendorf tubes with mixed vortex and smear-planted on a solid medium (blood agar plates), which split before and after the applications of disinfection.

### 3.3. Main and Subgroups

The main groups were derived from different concentrations of aqueous ozone, and NaOCl (positive control), and then subgroups were created according to the irrigation technique of each main group. First, NaOCl (positive control) group: Infected root canals were irrigated using a manual or ultrasonic technique for 180 s with 5.25% NaOCl. Second, 8 ppm aqueous ozone group: Aqueous ozone was obtained from a custom-made ozone generator (TeknO3zone, Izmir/Turkey) from the TeknO3zone Company. The aqueous ozone concentration of the distilled water was measured with a probe in the reactor tank and shown by a digital indicator on the generator. Infected root canals were irrigated with 8 mg/L aqueous ozone (TeknO3zone, Izmir/Turkey) by manual or ultrasonic technique for 180 seconds. Power control was maintained automatically by the automatic balancing system. Third, 12 ppm aqueous ozone group: Infected root canals were irrigated with 12 mg/L aqueous ozone (TeknO3zone, Izmir/Turkey) by manual or ultrasonic technique for 180 s. Fourth, 16ppm aqueous ozone group: Infected root canals were irrigated with 16 mg/L aqueous ozone (TeknO3zone, Izmir/Turkey) by manual or ultrasonic technique for 180 s.

### 3.4. Ultrasonic Technique Application

**Figure 1.**
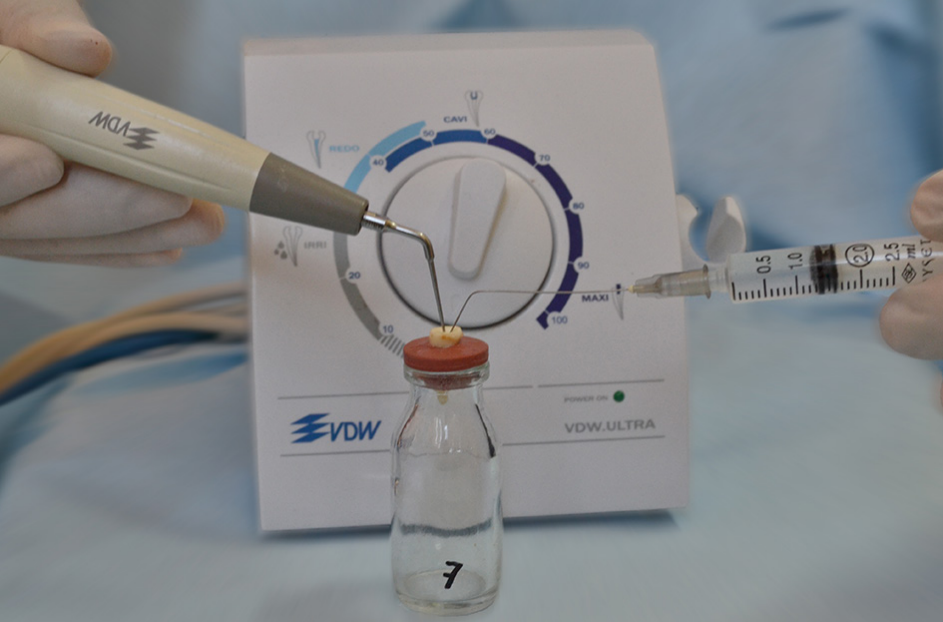
Aqueous Ozone With Ultrasonic Technique

Conventional irrigation technique simultaneously applied with ultrasonic vibration that produced from VDW.ULTRA (Satelec, Merignac Cedex, France) (Figure 1) device.

### 3.5. Antibacterial Evaluation

Root canals were contaminated with *E.* *faecalis* and left for 24 h. Paper points were placed in the root canals and waited 5 minutes to count the existence of bacteria both before and after root canal disinfection. The bacteria were counted before irrigation to ensure standardization, and then the examples whose values were under 1.5 X 108 CFU/mL were excluded. After the application of irrigation, CFU counts of the breeding colonies of microorganisms were performed on blood agar plates. Finally, the logs of the CFU counts were calculated.

### 3.6. Statistical Analysis

The obtained data of the irrigation solutions were analyzed using the SPSS statistical software program (version 14.0, SPSS Inc., Chicago, USA). The data were subjected to statistical analysis among four different irrigation solutions using 2-way analysis of variance (ANOVA). When significant differences were observed, Tukey post hoc test was applied to examine pairwise differences at a significance level of 0.05.

## 4. Results

The mean, standard deviation, and median values obtained after irrigation, as well as a statistical comparison of the log CFU mean values are shown in Table 1. As a result of this study, statistically significant differences were found between NaOCl and all aqueous ozone applied by manual technique (P < 0.05). Moreover, there were statistically significant differences among different concentrations of aqueous ozone with manual technique groups (P < 0.05). Statistically significant differences were not found between NaOCl and 16 ppm aqueous ozone applied with the ultrasonic technique (P > 0.05). Moreover, there were statistically significant differences between these two groups and the other aqueous ozone groups with the ultrasonic technique (P < 0.05). Although there were statistically significant differences between all aqueous ozone groups with the manual and ultrasonic techniques (P < 0.05), the NaOCl groups did not show statistically significant differences between the manual and ultrasonic techniques (P = 1.00).

**Table 1.** Mean (Standard Deviation) Together With Their Statistical Comparisons and Bacterial Reduction (%) Values That Were Obtained From all Groups ^a,b^

Groups	Manual Irrigation Technique		Ultrasonic Irrigation Technique	
	Mean (SD) (Log CFU mL^-1^)	Bacterial Reduction (%)	Mean (SD) (Log CFU mL^-1^)	Bacterial Reduction (%)
**NaOCl (Positive control)**	0.000 (0.00)	100%	0.000 (0.00)	100%
**8 ppm aqueous ozone **	3.060 (0.12)	62.6%	2.055 (0.12)	74.9%
**12 ppm aqueous ozone **	2.180 (0.15)	73.3%	1.045 (0.11)	87.2%
**16 ppm aqueous ozone**	0.700 (0.47)	91.4%	0.000 (0.00)	100%

^a^ CFU, colony-forming units; By the 2-way ANOVA, F = 780.721; P = 0.000 (P < 0.05).

^b^ Values with same superscript letter are statistically different at p < 0.05 by Tukey test.

## 5. Discussion

In endodontics, the removal of debris and elimination of remaining microorganisms are crucial for successful treatment of root canals. Lately, a few new trends of disinfectant agents like aqueous ozone, have been investigated against *E.* *faecalis*, which has shown resistance in root canals (7, 16, 17). However, aqueous ozone cannot achieve the same antibacterial effect as NaOCl, which is commonly preferred to use in root canal disinfection. Many researchers have already investigated the antimicrobial efficacy of various concentrations of NaOCl against resistant microorganisms (18-21). In particular; 5.25% NaOCl solution has shown the strongest bactericidal efficacy in eliminating all microorganisms in root canals and deeper dentinal tubules. Therefore, 5.25% NaOCl is recommended as an effective solution in the treatment of infected root canals due to its well-known antimicrobial effects (18-22). In the present study, we used 5.25% NaOCl in root canals as a positive control group. No wonder, when complete bacterial elimination was achieved, as indicated by aforementioned researches (18-22).

In general, one of the resistant microorganisms that has reduced endodontic treatment success and been isolated from root canals is *E. faecalis* (20-22). Therefore, we used this bacterium to obtain more realistic clinical results. In the past times, ozone has been studied as a new alternative disinfectant agent in root canals. Ozone has shown antimicrobial efficacy against resistant pathogens by neutralizing them or preventing their growth (7). In dentistry, ozone has been used in either gaseous or aqueous form to eliminate microorganisms in root canals (23, 24).

One study, evaluated the efficacy of aqueous ozone against *E.* *faecalis* in bovines. The root canals were irrigated with 4 mg/L aqueous ozone for 10 min. The root canal irrigation with aqueous ozone caused a considerable decrease in the amount of remaining bacteria (7). In another study, Hems et al. (15) examined the antibacterial effect of gaseous and aqueous ozone against *E.* *faecalis* in root canals. A significant reduction of the remaining bacteria was observed following the application of aqueous ozone for 240 s.

 Cardoso et al. (16) investigated the effectiveness of aqueous ozone to eradicate *E.* *faecalis* and *Candida albicans* from root canals. They demonstrated that aqueous ozone can eliminate bacteria. Furthermore, Estrela et al. (17) evaluated the antimicrobial efficacy of aqueous ozone and NaOCl in root canals inoculated with *E.* *faecalis*. Aqueous ozone was not achieved the complete elimination of *E.* *faecalis* after 20 minutes. In a recent study, Zan et al. (22) also investigated the antibacterial effect of 4 mg/L aqueous ozone against *E.* *faecalis* in root canals for 180 s. Although aqueous ozone showed a remarkable antibacterial effect, it did not show equal efficacy to that of the traditional NaOCl against *E.* *faecalis*.

 In the present study, the most significant difference regarding other studies (7, 15-17, 22) was the use of a higher aqueous ozone concentration (16 mg/L) with a manual irrigation technique. As a result, a significant reduction of *E.* *faecalis* was detected in the root canals. However, in spite of using higher concentration of aqueous ozone, complete disinfection was not achieved in human root canals. This result is similar to those of the aforementioned studies (7, 15-17, 22). A few studies have focused on the importance of irrigation solutions with an ultrasonic technique. For example, Nagayoshi et al. (7) examined the effect of aqueous ozone against *E.* *faecalis* and *Streptococcus mutans* in bovines. After aqueous ozone (4 mg/L) irrigation with an ultrasonic technique for 10 min, the viability of *E.* *faecalis* and *S. mutans* invading dentinal tubules significantly decreased.

 Moreover, aqueous ozone with an ultrasonic technique delivered the same antimicrobial activity as 2.5% NaOCl for 2 min. However, none of the solutions could achieve the complete elimination of bacteria. In another research, Arita et al. (8) investigated the antifungal effect of aqueous ozone (4 mg/L) combined with ultrasonication against *C. albicans* on acrylic resin plates. Although there was a slight reduction in the amount of fungi after 60 seconds, it took more than 30 minutes to achieve complete microbial elimination.

 In the present study, 16 ppm aqueous ozone in the ultrasonication group in 180 seconds achieved complete elimination of *E.* *faecalis* in root canals. This result may be due to the use of a different irrigation technique in the root canals (16 mg/L) than aforementioned studies (7, 8). Ultrasonication allows deeper penetration and effective agitation in dentin tubules and lateral canals, and as a result, increases the antibacterial efficacy of aqueous ozone in human root canals. Based on the results of the present study, a high concentration of aqueous ozone induced by an ultrasonic technique delivers the same antibacterial efficacy as 5.25% NaOCl for root canal disinfection. Consequently, aqueous ozone applied with an ultrasonic technique may be recommended as a disinfectant agent in endodontic treatments for its high bactericidal activity and suitable clinical irrigation time.
